# Error Analysis and Modeling of a Large Component Assembly Monitoring System Based on Multi-Sensor Fusion

**DOI:** 10.3390/s24185992

**Published:** 2024-09-15

**Authors:** Zhenggang Guo, Feng Lu, Tizhao Jiao, Jingqi Yu, Fu Chang

**Affiliations:** School of Mechanical Engineering, Dalian University of Technology of China, Dalian 116024, China; lulufeng@mail.dlut.edu.cn (F.L.);

**Keywords:** large components, hydroelectric generator rotor and stator, relative position in space, multi-sensor, error analysis and modeling

## Abstract

Large components are crucial in modern industrial applications, especially for internal gap monitoring and specific assembly methods. This paper examines the assembly of hydroelectric generator rotors and stators, introducing a spatial relative position monitoring system using multiple sensors. A dedicated position monitoring program is designed, and error sources within the system are thoroughly explored. Detailed error analysis and modeling reveal that verticality and angular errors significantly impact monitoring accuracy. To address this, two error control methods are proposed to effectively mitigate these issues, ensuring precise assembly of large components.

## 1. Introduction

Large components serve as the fundamental building blocks for numerous intricate industrial products, encompassing machinery, aerospace devices, and energy facilities. Their meticulous assembly is pivotal in ensuring the holistic functionality and performance of the end product. In practice, the assembly process constitutes a substantial portion of production, underscoring its indispensable significance [1]. As we transition into the era of high-performance industrial equipment, achieving high-precision design and processing of components has emerged as an unavoidable challenge [2]. In the assembly process of large components, enhancing efficiency and safety requires adopting a strategic approach to mitigate the impact of various assembly errors [3].

Due to the distinct characteristics of large-scale equipment, tailored assembly solutions are imperative to monitor errors in the assembly process effectively, thereby meeting stringent precision requirements [4,5,6]. To tackle the aforementioned assembly monitoring challenges, Liu et al. [7] propose an Intelligent Collaborative Assembly System (ICAS) that leverages a blend of data-driven methodologies and Augmented Reality (AR) technology to facilitate real-time monitoring of assembly processes for large and complex products; Pang et al. [8] proposed to utilize point cloud processing algorithms with digital twin (DT) technology to compose a physical twin scene (PT) to represent on-site assembly, and to compose a part-centered manual assembly-assisted monitoring system; Peng et al. [9] propose a remote measurement system that integrates attitude sensors and laser sensors to perform measurement tasks during docking in outdoor environments. This system aims to achieve geometric measurement modeling at each assembly stage of the component. Price et al. [10] suggested using laser scanning, encoders, and vision systems to construct a monitoring system aimed at improving lifting safety and efficiency. Fang et al. [11] introduced a vision-based load sway monitoring system to enhance crane lifting and lowering operations. Wu et al. [12] proposed a Bayesian Error Analysis and Reordering (BEAR) method based on the sequential Monte Carlo sampling algorithm, designed to analyze and correct errors systematically. Jiang et al. [13] propose a real-time quality monitoring prediction model for MMPs based on an error propagation network, aimed at ensuring the robustness of the machining process and enhancing machining quality. Yang et al. [14] propose targeting laser radiation-induced gases and introducing a functional platform aimed at systematically controlling functional gradient structures and modeling architectural aspects in the build direction during additive manufacturing.

**Figure 1:**
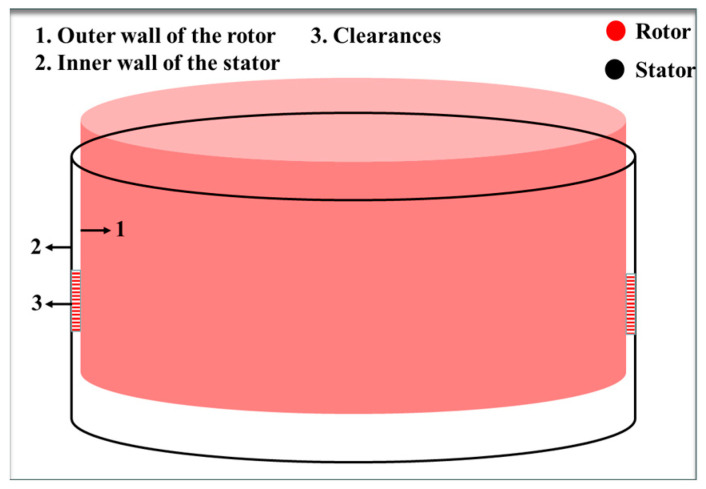
Relative position of rotor and stator.

Various assembly monitoring strategies have been proposed in the literature, such as combining data-driven approaches with augmented reality (AR), employing point cloud processing algorithms with digital twin (DT) technology, and integrating attitude sensors with laser sensors. These methods are applicable to diverse assembly scenarios and offer substantial insights for this study. However, specific working conditions require monitoring internal gaps in large components, like the assembly process of hydroelectric generator rotors and stators [15,16,17]. This necessitates real-time monitoring of the horizontal distance between the rotor’s outer wall and the stator’s inner wall, as shown in Figure 1. This complicates the external establishment of location relationships necessary to meet monitoring requirements which current monitoring systems may not adequately address. Furthermore, there is a need to develop an error model tailored to the rotor and stator assembly process using the error analysis and modeling methods discussed in existing literature. To enhance the assembly accuracy of hydro generator rotor and stator assemblies, a rotor lifting relative position monitoring system is proposed based on previous research methods. Additionally, a mapping model is established to correlate 24 system errors with assembly errors [18]. The accuracy and effectiveness of this error model are validated through comprehensive data analysis, providing a solid theoretical foundation for the position monitoring system. 

## 2. Rotor Lifting Monitoring Program

### 2.1. Analysis of Rotor Lifting Process

The hydroelectric generator rotor is significantly large, requiring two sets of bridge cranes with articulated lifting shafts and lower hanging balance beams. Numerous ground staff and crane operators must coordinate closely. The cranes lift the rotor from its installation point to directly above the pit (stator), and then lower it into place. During the assembly, it is crucial to continuously monitor the gap between the rotor and stator to ensure it meets assembly specifications. Achieving millimeter-level accuracy is essential [19]. Given the rotor’s size, maintaining this precision over a ten-meter spatial scale is challenging. Accurately monitoring their relative positions demands advanced techniques and meticulous attention to detail.

For the assembly of the hydro generator rotor and stator, a spatial relative position monitoring system is designed to ensure precision. This system includes ultrasonic range sensors, laser range sensors, a data collector [20,21] and host computer interface (HMI) [22]. Ultrasonic and laser sensors are utilized to gather spatial position data of the rotor. This data is collected and integrated by a data collector. The host computer program then processes the collected data to determine the relative spatial position between the rotor and the stator. Finally, the host computer visualizes this positional relationship. The critical checkpoint in the assembly process is the horizontal distance between the outer wall of the rotor and the inner wall of the stator. The position monitoring system must not interfere with the assembly process itself. Therefore, in the design phase, direct access to data from the assembly checkpoint is not feasible. This discrepancy necessitates the development of a mathematical model to convert between the sensing monitoring points and the assembly checkpoints [23]. Through this model, various errors inherent in the system can be analyzed and mitigated, ensuring the accuracy of the position monitoring system.

### 2.2. Principle of Position Monitoring

In the attitude monitoring system, several parameters are scrutinized, such as the rotor’s ground clearance, the gap between the rotor’s outer wall and the stator’s inner wall, and the spatial orientation of the rotor. To achieve real-time and precise monitoring of these parameters, the system integrates advanced technologies including ultrasonic ranging, laser ranging, and microcontroller-based measurement and control technology [24]. This comprehensive integration facilitates the monitoring and analysis of the relative positioning between the rotor and stator.

In this system, the height of the rotor from the ground is determined using a laser distance measuring sensor. Laser distance measurement technology is renowned for its exceptional positioning accuracy and stability, particularly in long-distance measurements, where it maintains high accuracy even in complex environments [25,26]. Given the substantial height of the rotor from the ground and the requirement for stable, long-term monitoring, the superior performance of the laser ranging sensor provides a reliable measurement solution for the system. During the assembly process, the gap between the rotor’s outer wall and the stator’s inner wall is a critical parameter requiring sensitive monitoring. To address this, an ultrasonic ranging sensor was selected. Ultrasonic ranging technology utilizes ultrasonic waves for measurement, covering a circular detection area [27,28]. Its emission waveform, resembling a waist drum, is ideally suited to mitigate the impact of the stator’s inner wall gap on monitoring accuracy.

**Figure 2:**
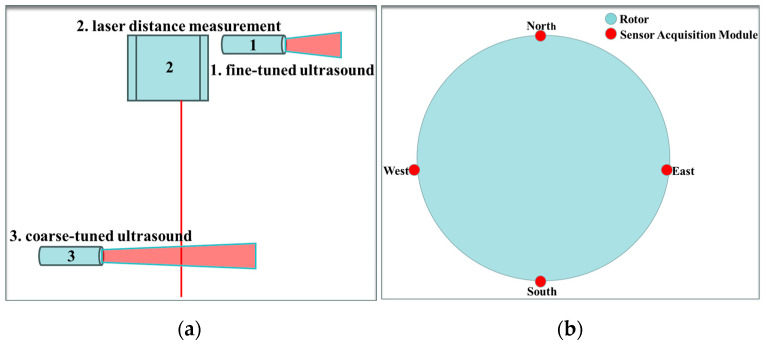
Sensor module arrangement diagram: (**a**) multi-sensor arrangements; (**b**) sensor acquisition module arrangements.

The synergy among multiple sensors in this system plays a pivotal role in enhancing the assembly accuracy of the rotor and stator. In the design of the sensor acquisition module, one laser ranging sensor is oriented vertically, while two ultrasonic ranging sensors are strategically deployed horizontally: one for coarse adjustment and the other for fine adjustment, as shown in Figure 2a. Along the outer circumference of the rotor, four sets of sensor acquisition modules are uniformly distributed, as shown in Figure 2b.

**Figure 3:**
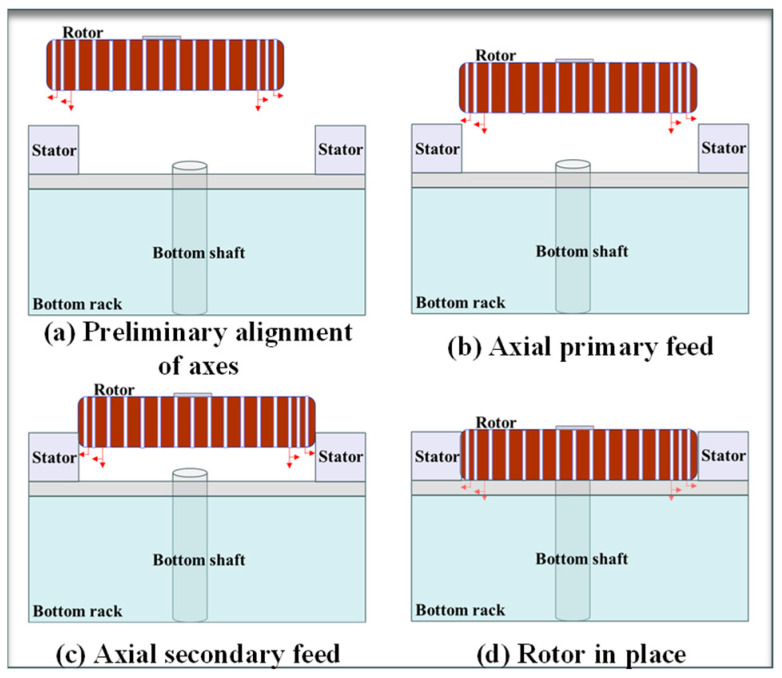
Rotor lifting process.

This monitoring program divides the spatial relative position monitoring of the hydro generator rotor and stator assembly into four distinct steps: preliminary axial alignment, axial primary feeding, axial secondary feeding, and rotor positioning, as illustrated in Figure 3. During these steps, the laser ranging sensor is utilized for preliminary axial alignment, where its ground probing data determines whether the rotor enters the lifting area. The coarse-adjusted ultrasonic ranging sensor and the fine-adjusted ultrasonic ranging sensor monitor the gap between the rotor and the stator during the two axial feeding processes, ensuring precise coordination between the rotor and the stator.

**Figure 4:**
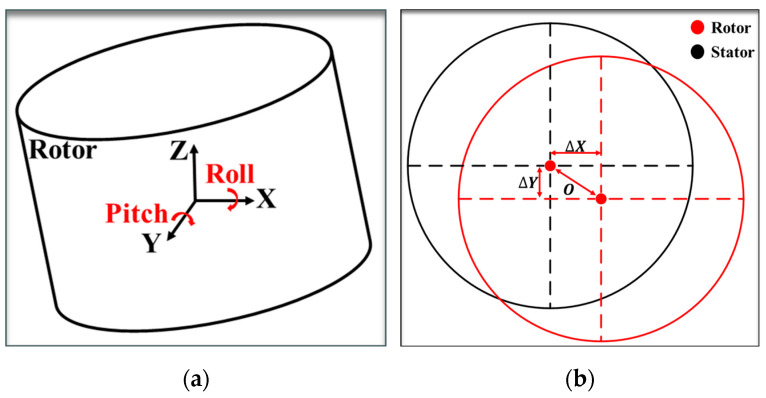
Spatial relative orientations of rotor and stator: (**a**) spatial attitude of the rotor; (**b**) relative position of rotor and pinned stator.

The relative positions of the rotor and stator in space are determined from the collected data of multiple sensors. This includes calculating the pitch angle (pitch) and the roll angle (roll) of the rotor [29], as shown in Figure 4a; the relative positions of the rotor and the stator $∆x_{1}$, $∆y_{1}$ and the centroidal distance $O_{1}$ in the axial primary feeding stage; and the relative positions of the rotor and the stator $∆x_{2}$, $∆y_{2}$ and the centroidal distance $O_{2}$ in the axial secondary feeding stage, as shown in Figure 4b. We calculate the spatial attitude angle of the rotor by using an inverse trigonometric function based on the numerical difference between the laser sensors and the rotor’s diameter.

(1) $$pitch=\sin^{-1}\frac{h_{1}-h_{3}}{18433-2L_{1}}$$

(2) $$roll=\sin^{-1}\frac{h_{4}-h_{2}}{18433-2L_{1}}$$

In the formula: $h_{1}$, $h_{2}$, $h_{3}$, $h_{4}$ is the height of the four groups from the ground at the four equal points of the maximum outer diameter of the rotor; 18,433 mm is the maximum outer diameter of the rotor; $L_{1}$ is the horizontal distance between the probes of the four groups of laser ranging sensors and the corresponding rotor at the four equal points of the maximum outer diameter.

(3) $$∆x_{1}=L_{23}-L_{21}$$

(4) $$∆y_{1}=L_{24}-L_{22}$$

(5) $$O_{1}=\sqrt{{∆x_{1}}^{2}+{∆y_{1}}^{2}}$$

In the formula: $∆x_{1}$, $∆y_{1}$ is the distance between the center of the rotor circle and the center of the stator circle in the X, Y direction during the axial one-time feeding process; $O_{1}$ is the centroidal distance between the rotor and the stator during the axial one-time feeding process; $L_{21}$, $L_{22}$, $L_{23}$, $L_{24}$ is the collected data from the coarse-adjustment ultrasonic transducer in the four directions of east, south, west, and north during the axial one-time feeding process.

(6) $$∆x_{2}=L_{33}-L_{31}$$

(7) $$∆y_{2}=L_{34}-L_{32}$$

(8) $$O_{2}=\sqrt{{∆x_{2}}^{2}+{∆y_{2}}^{2}}$$

In the formula: $∆x_{2}$, $∆y_{2}$ is the distance between the center of the rotor circle and the center of the stator circle in the X, Y direction during the axial secondary feeding process; $O_{2}$ is the centroidal distance between the rotor and the stator during the axial secondary feeding process; $L_{31}$, $L_{32}$, $L_{33}$, $L_{34}$ is the collected data from the finely tuned ultrasonic transducers in the four directions of east, south, west, and north during the axial secondary feeding process.

## 3. Error Analysis and Modeling of Position Monitoring Systems

### 3.1. Error Source Analysis

In this paper, the spatial relative position monitoring system for the rotor and stator during assembly relies on integrated measurements from multiple sensor devices. While a multi-sensor configuration enhances the comprehensiveness and accuracy of measurement data, practical applications are susceptible to installation and calibration errors influenced by human factors [30]. These errors can disrupt the precise monitoring of rotor-stator relative positions and their exact alignment, potentially heightening the risk of rotor-stator collisions.

**Figure 5:**
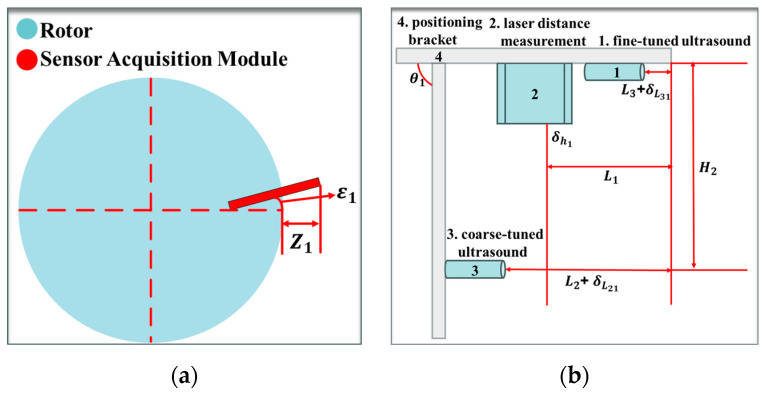
Distribution of errors: (**a**) installation errors in the sensor acquisition module; (**b**) multi-sensor mounting errors.

As shown in Figure 5a,b, in this system, the four coarse-tuned ultrasonic ranging sensors have the localization errors of $\delta_{L_{21}}$, $\delta_{L_{22}}$, $\delta_{L_{23}}$, $\delta_{L_{24}}$, respectively; the four fine-tuned ultrasonic ranging sensors have the localization errors of $\delta_{L_{31}}$, $\delta_{L_{32}}$, $\delta_{L_{33}}$, $\delta_{L_{34}}$, respectively; the four laser ranging sensors have measurement errors of $\delta_{h_{1}}$, $\delta_{h_{2}}$, $\delta_{h_{3}}$, $\delta_{h_{4}}$, respectively; the four sensor acquisition modules have angular errors of $\varepsilon_{1}$, $\varepsilon_{2}$, $\varepsilon_{3}$, $\varepsilon_{4}$ in the positive direction of their counterparts, respectively; and the horizontal bars of the brackets of the four sensor acquisition modules have perpendicularity errors of $\theta_{1}$, $\theta_{2}$, $\theta_{3}$, $\theta_{4}$ with respect to the vertical bars; There are calibration errors of $Z_{1}$, $Z_{2}$, $Z_{3}$, $Z_{4}$ between the origin of the four sensor acquisition modules and the outer edge of the rotor.

In summary, the spatial relative attitude monitoring system of the rotor involves a comprehensive set of 24 errors, meticulously documented in Table 1. Throughout the assembly process of the rotor and stator, these errors result from the intricacies of installing and positioning multiple sensors and the sensor acquisition module. They collectively exert a profound influence on the precision of attitude monitoring, thereby crucially impacting the accuracy of the assembly process. These influences may derive from the cumulative, interactive, or compounded effects of these errors [31], necessitating meticulous consideration and adjustment at each stage of rotor assembly to ensure the exactitude and dependability of the final assembly outcomes.

### 3.2. Error Modeling

#### 3.2.1. Modeling of Errors during Axial Primary Feeds

**Figure 6:**
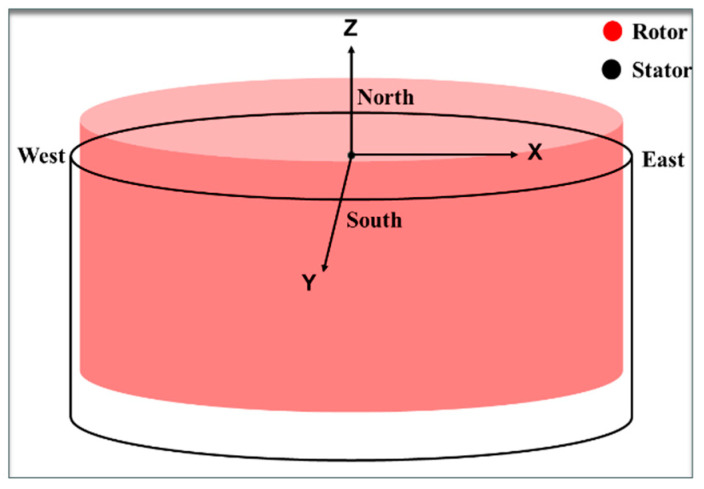
Error model coordinate system.

Based on the results of the error source analysis in the position monitoring system, a spatial assembly coordinate system is established with the hydro generator stator’s main axis as the coordinate origin. The east direction is defined as the X-direction, the south direction as the Y-direction, and the vertical upward direction as the Z-direction [32], as shown in Figure 6. A chi-square coordinate transformation is used to model the errors in the rotor assembly process [33]. Taking the axial feeding process of the rotor as an example, we analyze the degree of influence of each error on the assembly accuracy.

(1) Rotor points due east:

In the relative position monitoring error of the rotor’s due east point, its main error-sensitive parameter is in the X coordinate axis. The sensitive error sources include the positioning error $\delta_{L_{21}}$ of the coarse-adjusted ultrasonic ranging sensor, the calibration error $Z_{1}$ between the origin of the sensor acquisition module and the outer edge of the rotor, the perpendicularity error $\theta_{1}$ between the horizontal bar and the vertical bar of the support of the sensor acquisition module, and the angular error $\varepsilon_{1}$ between the sensor acquisition module and its corresponding positive direction, the error components in the X, Y, Z directions are represented by the following expressions.

(9) $$\alpha_{1}=\left[\begin{array}{cccc} 1 & 1 & H_{2} & L_{2} \end{array}\right]\times \left[\begin{array}{c} \delta_{L_{21}}\\ Z_{1}\\ \sin\theta_{1}\\ 1-\cos\varepsilon_{1} \end{array}\right]$$

(10) $$\beta_{1}=L_{2}\times \sin\varepsilon_{1}$$

(11) $$\gamma_{1}=\delta_{h_{1}}$$

In the formula: $\alpha_{1}$, $\beta_{1}$, $\gamma_{1}$ are the monitoring errors of the rotor’s positive east direction point in the X, Y, Z directions under the space assembly coordinate system, respectively.

(2) Rotor points due south:

In the relative position monitoring error of the rotor at the point due south, its main error-sensitive parameter is in the Y coordinate axis. The sensitive error sources include the positioning error $\delta_{L_{22}}$ of the coarse-adjusted ultrasonic ranging sensor, the calibration error $Z_{2}$ between the origin of the sensor acquisition module and the outer edge of the rotor, the perpendicularity error $\theta_{2}$ between the horizontal bar and the vertical bar of the holder of the sensor acquisition module, and the angular error $\varepsilon_{2}$ between the sensor acquisition module and its corresponding positive direction, the error components in the X, Y, Z directions are represented by the following expressions.

(12) $$\alpha_{2}=L_{2}\times \sin\varepsilon_{2}$$

(13) $$\beta_{2}=\left[\begin{array}{cccc} 1 & 1 & H_{2} & L_{2} \end{array}\right]\times \left[\begin{array}{c} \delta_{L_{22}}\\ Z_{2}\\ \sin\theta_{2}\\ 1-\cos\varepsilon_{2} \end{array}\right]$$

(14) $$\gamma_{2}=\delta_{h_{2}}$$

In the formula: $\alpha_{2}$, $\beta_{2}$, $\gamma_{2}$ are the monitoring errors of the rotor’s due south direction point in the X, Y, Z directions under the space assembly coordinate system, respectively.

(3) Rotor points due west:

The main error-sensitive parameters in the relative position monitoring error at the rotor’s due west point are in the X coordinate axis. The sensitive error sources include the positioning error $\delta_{L_{23}}$ of the coarse-adjusted ultrasonic ranging sensor, the calibration error $Z_{3}$ between the origin of the sensor acquisition module and the outer edge of the rotor, the perpendicularity error $\theta_{3}$ between the horizontal bar and the vertical bar of the support of the sensor acquisition module, and the angular error $\varepsilon_{3}$ between the sensor acquisition module and its corresponding positive direction, the error components in the X, Y, Z directions are represented by the following expressions.

(15) $$\alpha_{3}=\left[\begin{array}{cccc} 1 & 1 & H_{2} & L_{2} \end{array}\right]\times \left[\begin{array}{c} \delta_{L_{23}}\\ Z_{3}\\ \sin\theta_{3}\\ 1-\cos\varepsilon_{3} \end{array}\right]$$

(16) $$\beta_{3}=L_{2}\times \sin\varepsilon_{3}$$

(17) $$\gamma_{3}=\delta_{h_{3}}$$

In the formula: $\alpha_{3}$, $\beta_{3}$, $\gamma_{3}$ are the monitoring errors in X, Y, Z directions of the rotor’s positive westward point in the spatial assembly coordinate system, respectively.

(4) Rotor points due north:

In the relative position monitoring error of the rotor’s due north point, its main error-sensitive parameter is in the Y coordinate axis. The sensitive error sources include the positioning error $\delta_{L_{24}}$ of the coarse-adjusted ultrasonic ranging sensor, the calibration error $Z_{4}$ between the origin of the sensor acquisition module and the outer edge of the rotor, the perpendicularity error $\theta_{4}$ between the horizontal bar and the vertical bar of the holder of the sensor acquisition module, and the angular error $\varepsilon_{4}$ between the sensor acquisition module and its corresponding positive direction, the error components in the X, Y, Z directions are represented by the following expressions.

(18) $$\alpha_{4}=L_{2}\times \sin\varepsilon_{4}$$

(19) $$\beta_{4}=\left[\begin{array}{cccc} 1 & 1 & H_{2} & L_{2} \end{array}\right]\times \left[\begin{array}{c} \delta_{L_{24}}\\ Z_{4}\\ \sin\theta_{4}\\ 1-\cos\varepsilon_{4} \end{array}\right]$$

(20) $$\gamma_{4}=\delta_{h_{4}}$$

In the formula: $\alpha_{4}$, $\beta_{4}$, $\gamma_{4}$ are the monitoring errors of the rotor’s due north direction point in the X, Y, Z directions under the space assembly coordinate system, respectively.

In summary, during the axial feeding process of the rotor, there will be errors in the gap monitoring at the four equidistant points along the outermost edge of the rotor. These errors collectively impact the overall assembly accuracy of the rotor. The specific expression is as follows.

(21) $$\left[\begin{array}{ccc} \delta_{x} & \delta_{y} & \delta_{z} \end{array}\right]=\left[\begin{array}{cccc} 1 & 1 & 1 & 1 \end{array}\right]\times \left[\begin{array}{ccc} \alpha_{1} & \beta_{1} & \gamma_{1}\\ \alpha_{2} & \beta_{2} & \gamma_{2}\\ \alpha_{3} & \beta_{3} & \gamma_{3}\\ \alpha_{4} & \beta_{4} & \gamma_{4} \end{array}\right]$$

(22) $$O=\sqrt{{\delta_{x}}^{2}+{\delta_{y}}^{2}}$$

In the formula: $\delta_{z}$ is the deviation of the rotor from the Z-axis in the spatial assembly coordinate system; $\delta_{x}$ is the deviation of the rotor from the X-axis in the spatial assembly coordinate system; $\delta_{y}$ is the deviation of the rotor from the Y-axis in the spatial assembly coordinate system; and O is the deviation of the rotor from the centroidal distance of the XY plane in the spatial coordinate system.

#### 3.2.2. Modeling of Errors during Axial Secondary Feeds

In the axial secondary feeding process of the rotor, which is a critical stage preceding the assembly of the rotor and stator, the finely tuned ultrasonic sensors exclusively fulfill a monitoring role. Therefore, attention is primarily directed towards potential errors stemming from the installation and positioning of these sensors, along with considerations for the sensor acquisition module. These error sources are meticulously modeled to assess their impact on the rotor within the spatial assembly coordinate system.

(1) Rotor points due east:

At the eastern point of the rotor, its primary error-sensitive parameter lies along the X coordinate axis. Key error sources include the positioning error $\delta_{L_{31}}$ of the finely tuned ultrasonic ranging sensor, the calibration error $Z_{1}$ between the origin of the sensor acquisition module and the outer edge of the rotor, and the angular error $\varepsilon_{1}$ between the sensor acquisition module and its corresponding positive direction. The error components in the X, Y directions are shown in the following equation.

(23) $$\overset{´}{\alpha_{1}}=\left[\begin{array}{ccc} 1 & 1 & L_{3} \end{array}\right]\times \left[\begin{array}{c} \delta_{L_{31}}\\ Z_{1}\\ 1-\cos\varepsilon_{1} \end{array}\right]$$

(24) $$\overset{´}{\beta_{1}}=L_{3}\times \sin\varepsilon_{1}$$

In the formula: $\overset{´}{\alpha_{1}}$, $\overset{´}{\beta_{1}}$ are the monitoring errors of the rotor’s positive east direction point in the X, Y directions under the space assembly coordinate system, respectively.

(2) Rotor points due south:

At the point due south of the rotor, the primary error-sensitive parameter lies along the Y coordinate axis. Critical error sources include the positioning error $\delta_{L_{32}}$ of the finely tuned ultrasonic ranging sensor, the calibration error $Z_{2}$ between the origin of the sensor acquisition module and the outer edge of the rotor, and the angular error $\varepsilon_{2}$ between the sensor acquisition module and its corresponding positive direction. The error components in the X, Y directions are shown in the following equation.

(25) $$\overset{´}{\alpha_{2}}=L_{3}\times \sin\varepsilon_{2}$$

(26) $$\overset{´}{\beta_{2}}=\left[\begin{array}{ccc} 1 & 1 & L_{3} \end{array}\right]\times \left[\begin{array}{c} \delta_{L_{32}}\\ Z_{2}\\ 1-\cos\varepsilon_{2} \end{array}\right]$$

In the formula: $\overset{´}{\alpha_{2}}$, $\overset{´}{\beta_{2}}$ are the monitoring errors of the rotor’s positive south direction point in the X, Y directions under the space assembly coordinate system, respectively.

(3) Rotor points due west:

At the point westward of the rotor, the primary error-sensitive parameter resides along the X coordinate axis. Critical error sources encompass the positioning error $\delta_{L_{33}}$ of the finely tuned ultrasonic ranging sensor, the calibration error $Z_{3}$ between the origin of the sensor acquisition module and the outer edge of the rotor, and the angular error $\varepsilon_{3}$ between the sensor acquisition module and its corresponding positive direction. The error components in the X, Y directions are shown in the following equation.

(27) $$\overset{´}{\alpha_{3}}=\left[\begin{array}{ccc} 1 & 1 & L_{3} \end{array}\right]\times \left[\begin{array}{c} \delta_{L_{33}}\\ Z_{3}\\ 1-\cos\varepsilon_{3} \end{array}\right]$$

(28) $$\overset{´}{\beta_{3}}=L_{3}\times \sin\varepsilon_{3}$$

In the formula: $\overset{´}{\alpha_{3}}$, $\overset{´}{\beta_{3}}$ are the monitoring errors in the X, Y directions of the rotor’s positive westward point in the spatial assembly coordinate system, respectively.

(4) Rotor points due north:

At the northern point of the rotor, the primary error-sensitive parameter pertains to the Y coordinate axis. Significant error sources encompass the positioning error $\delta_{L_{34}}$ of the finely tuned ultrasonic ranging sensor, the calibration error $Z_{4}$ between the origin of the sensor acquisition module and the outer edge of the rotor, and the angular error $\varepsilon_{4}$ between the sensor acquisition module and its corresponding positive direction. The error components in the X, Y directions are shown in the following equation.

(29) $$\overset{´}{\alpha_{4}}=L_{3}\times \sin\varepsilon_{4}$$

(30) $$\overset{´}{\beta_{4}}=\left[\begin{array}{ccc} 1 & 1 & L_{3} \end{array}\right]\times \left[\begin{array}{c} \delta_{L_{34}}\\ Z_{4}\\ 1-\cos\varepsilon_{4} \end{array}\right]$$

In the formula: $\overset{´}{\alpha_{4}}$, $\overset{´}{\beta_{4}}$ are the monitoring errors of the rotor’s due north direction point in the X, Y directions under the space assembly coordinate system, respectively.

In summary, during the axial secondary feeding of the rotor, errors in gap monitoring occur at all four equidistant points on the outermost edge of the rotor. These errors are delineated as follows.

(31) $$\left[\begin{array}{cc} \overset{´}{\delta_{x}} & \overset{´}{\delta_{y}} \end{array}\right]=\left[\begin{array}{cccc} 1 & 1 & 1 & 1 \end{array}\right]\times \left[\begin{array}{cc} \overset{´}{\alpha_{1}} & \overset{´}{\beta_{1}}\\ \overset{´}{\alpha_{2}} & \overset{´}{\beta_{2}}\\ \overset{´}{\alpha_{3}} & \overset{´}{\beta_{3}}\\ \overset{´}{\alpha_{4}} & \overset{´}{\beta_{4}} \end{array}\right]$$

(32) $$\overset{´}{O}=\sqrt{{\overset{´}{\delta_{x}}}^{2}+{\overset{´}{\delta_{y}}}^{2}}$$

In the formula: $\overset{´}{\delta_{x}}$ and $\overset{´}{\delta_{y}}$ represent the errors of the rotor along the X-axis and Y-axis directions in the spatial assembly coordinate system, respectively; and $\overset{´}{O}$ denotes the deviation in the centroidal distance of the rotor from the XY plane within the spatial coordinate system.

## 4. Model Validation and Analysis

### 4.1. Model Verification

**Figure 7:**
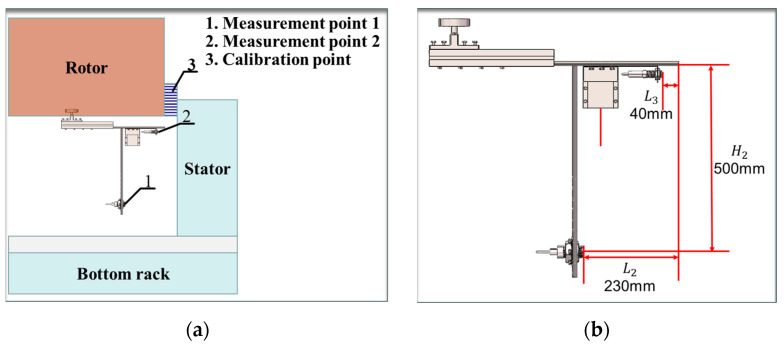
Schematic diagram of the experiment: (**a**) sensor acquisition module installation schematic; (**b**) relative position of multiple sensors.

Based on the provided research content, the bracket design and sensor fitting were refined, followed by experimental validation after installation at the bottom of the rotor in a specific configuration, as illustrated in Figure 7a. In this setup, the sensor bracket is affixed to the rotor’s underside using a magnetic mount, while the sensor acquisition module’s origin is precisely aligned with the rotor’s outer edge using a calibration plate. In Figure 7a, “measurement point 1” denotes the distance of the roughly adjusted ultrasonic ranging sensor from the stator’s inner wall, “measurement point 2” represents the distance of the finely adjusted ultrasonic ranging sensor from the stator’s inner wall, and the "calibration point" signifies the distance between the outer wall of the rotor and the stator’s inner wall.

As shown in Figure 7b, in this experiment, the design size of $H_{2}$ is 500 mm, the design size of $L_{2}$ is 230 mm, and the design size of $L_{3}$ is 40 mm. The degree of influence of the above error sources on the monitoring system is now analyzed sequentially by the control variable method with the following parameters.

#### 4.1.1. Axial Primary Feed Process

In the assembly process of a hydroelectric generator’s rotor and stator, the required assembly index mandates that the gap between the two components be within 20 mm. However, during the axial feeding stage, only the sensor acquisition module is initially lowered into the assembly monitoring area, while the rotor remains above this area. Therefore, the error margin for monitoring can be set to 30 mm to facilitate real-time adjustments of the rotor. The quantitative analysis of each error source’s influence on the monitoring system is conducted using its respective error model equations (Formula (7)–(22)). For this analysis, the distance classification error is defined as 10mm, and the angular classification error is defined as 10°, facilitating precise calculation and analysis. 

(1) Assuming that the system consists only 10 mm of positioning error.

$$\delta_{x}=\delta_{L_{21}}+\delta_{L_{23}}=20,\;\delta_{y}=\delta_{L_{22}}+\delta_{L_{24}}=20,\;\delta_{z}=0.$$

$$\left[\begin{array}{ccc} \delta_{x} & \delta_{y} & \delta_{z} \end{array}\right]=\left[\begin{array}{ccc} 20 & 20 & 0 \end{array}\right],\;O=\sqrt{20^{2}+20^{2}}=28.28.$$

(2) Assuming that the system has a measurement error of only 10 mm.

$$\delta_{x}=0,\;\delta_{y}=0,\;\delta_{z}=\delta_{h_{1}}+\delta_{h_{2}}+\delta_{h_{3}}+\delta_{h_{4}}=40.$$

$$\left[\begin{array}{ccc} \delta_{x} & \delta_{y} & \delta_{z} \end{array}\right]=\left[\begin{array}{ccc} 0 & 0 & 40 \end{array}\right],\;O=0.$$

(3) Assuming that the system only has a calibration error of 10 mm.

$$\delta_{x}=Z_{1}+Z_{3}=20,\;\delta_{y}=Z_{2}+Z_{4}=20,\;\delta_{z}=0.$$

$$\left[\begin{array}{ccc} \delta_{x} & \delta_{y} & \delta_{z} \end{array}\right]=\left[\begin{array}{ccc} 20 & 20 & 0 \end{array}\right],\;O=\sqrt{20^{2}+20^{2}}=28.28.$$

(4) Assuming that the system has an angular error of only 10°.

$$\delta_{x}=L_{2}\times (1-\cos\varepsilon_{1})+L_{2}\times \sin\varepsilon_{2}+L_{2}\times (1-\cos\varepsilon_{3})+L_{2}\times \sin\varepsilon_{4}=86.87.$$

$$\delta_{y}=L_{2}\times \sin\varepsilon_{1}+L_{2}\times (1-\cos\varepsilon_{2})+L_{2}\times \sin\varepsilon_{3}+L_{2}\times (1-\cos\varepsilon_{4})=86.87$$

$$\delta_{z}=0,\;\left[\begin{array}{ccc} \delta_{x} & \delta_{y} & \delta_{z} \end{array}\right]=\left[\begin{array}{ccc} 86.87 & 86.87 & 0 \end{array}\right],\;O=\sqrt{86.87^{2}+86.87^{2}}=122.84.$$

(5) Assuming that the system has a verticality error of only 10°.

$$\delta_{x}=H_{2}\times \sin\theta_{1}+H_{2}\times \sin\theta_{3}=174.$$

$$\delta_{y}=H_{2}\times \sin\theta_{2}+H_{2}\times \sin\theta_{4}=174$$

$$\delta_{z}=0,\;\left[\begin{array}{ccc} \delta_{x} & \delta_{y} & \delta_{z} \end{array}\right]=\left[\begin{array}{ccc} 174 & 174 & 0 \end{array}\right]=\sqrt{174^{2}+174^{2}}=246.07$$

#### 4.1.2. Axial Secondary Feed Process

In the axial secondary feeding stage, both the sensor acquisition module and the rotor have been lowered into the detection area. At this point, the system’s detection accuracy must be strictly ensured. This means maintaining consistency between the system’s error range and the actual rotor assembly, which must remain within 20 mm. Each error source’s influence on the monitoring system is meticulously analyzed using its respective error model equations (Formula (23)–(32)). Throughout the analysis, the distance categorization error remains defined at 10 mm, and the angular categorization error at 10°, ensuring streamlined calculation and analysis processes.

(1) Assuming that the system has only 10 mm of positioning error.

$$\overset{´}{\delta_{x}}=\delta_{L_{31}}+\delta_{L_{33}}=20,\;\overset{´}{\delta_{y}}=\delta_{L_{32}}+\delta_{L_{34}}=20,\;\delta_{z}=0.$$

$$\left[\begin{array}{ccc} \overset{´}{\delta_{x}} & \overset{´}{\delta_{y}} & \delta_{z} \end{array}\right]=\left[\begin{array}{ccc} 20 & 20 & 0 \end{array}\right],\;\overset{´}{O}=\sqrt{20^{2}+20^{2}}=28.28.$$

(2) Assuming that the system has a measurement error of only 10 mm.

$$\overset{´}{\delta_{x}}=0,\;\overset{´}{\delta_{y}}=0,\;\delta_{z}=\delta_{h_{1}}+\delta_{h_{2}}+\delta_{h_{3}}+\delta_{h_{4}}=40.$$

$$\left[\begin{array}{ccc} \overset{´}{\delta_{x}} & \overset{´}{\delta_{y}} & \delta_{z} \end{array}\right]=\left[\begin{array}{ccc} 0 & 0 & 40 \end{array}\right],\;\overset{´}{O}=0.$$

(3) Assuming that the system only has a calibration error of 10 mm.

$$\overset{´}{\delta_{x}}=Z_{1}+Z_{3}=20,\;\overset{´}{\delta_{y}}=Z_{2}+Z_{4}=20,\;\delta_{z}=0.$$

$$\left[\begin{array}{ccc} \overset{´}{\delta_{x}} & \overset{´}{\delta_{y}} & \delta_{z} \end{array}\right]=\left[\begin{array}{ccc} 20 & 20 & 0 \end{array}\right],\;\overset{´}{O}=\sqrt{20^{2}+20^{2}}=28.28.$$

(4) Assuming that the system has an angular error of only 10°.

$$\overset{´}{\delta_{x}}=L_{3}\times (1-\cos\varepsilon_{1})+L_{3}\times \sin\varepsilon_{2}+L_{3}\times (1-\cos\varepsilon_{3})+L_{3}\times \sin\varepsilon_{4}=15.11.$$

$$\overset{´}{\delta_{y}}=L_{3}\times \sin\varepsilon_{1}+L_{3}\times (1-\cos\varepsilon_{2})+L_{3}\times \sin\varepsilon_{3}+L_{3}\times (1-\cos\varepsilon_{4})=15.11.$$

$$\delta_{z}=0,\;\left[\begin{array}{ccc} \overset{´}{\delta_{x}} & \overset{´}{\delta_{y}} & \delta_{z} \end{array}\right]=\left[\begin{array}{ccc} 15.11 & 15.11 & 0 \end{array}\right],\;\overset{´}{O}=\sqrt{15.11^{2}+15.11^{2}}=21.37.$$

**Figure 8:**
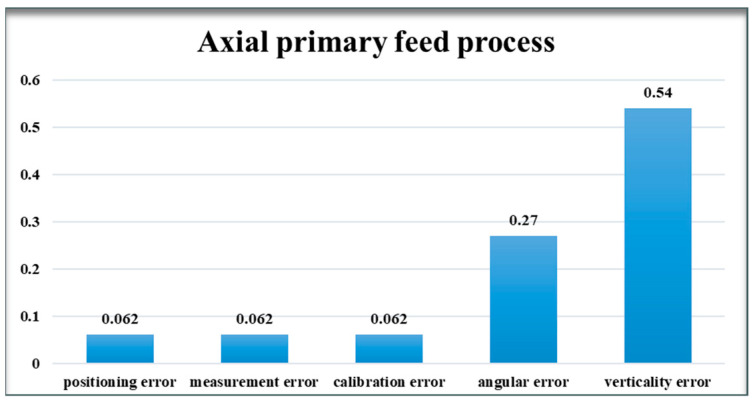
Axial primary feed process.

**Figure 9:**
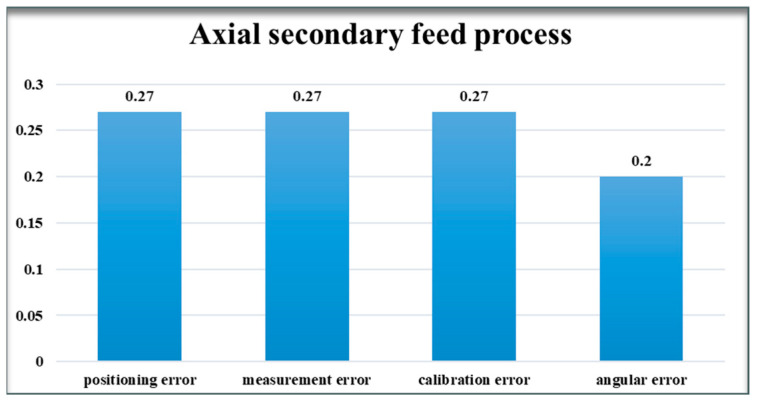
Axial secondary feed process.

Analyzing and comparing the results of the cumulative effect of the aforementioned errors on the relative position of the rotor and stator allows us to ascertain the degree of influence of each error type on the position monitoring system, as depicted in Figure 8 and Figure 9.

### 4.2. Model Analysis

After the above analysis of errors and modeling results, it can be seen that many error sources in the position monitoring system will have a greater or lesser impact on the accuracy of the system. In the axial primary feed stage, the influence of perpendicularity error is relatively large, followed by angular error, while the influence of the rest of the errors is low. This is due to the fact that the coarse tuning ultrasonic sensor plays a major role in the axial primary feed stage. The coarse tuning ultrasonic sensor is up to 500 mm away from the bottom of the rotor and up to 230 mm away from the outer wall of the rotor, which results in a large error component in the direction of the coordinate axes when there are perpendicularity errors and angular errors. In the axial secondary feeding stage, the angular error accounts for a smaller proportion, because it is the fine-tuned ultrasonic sensor that plays a major role in the axial secondary feed phase. The distance of the fine-tuned ultrasonic sensor from the rotor wall is only 40 mm, which does not generate large error components in the direction of the axes. Therefore, we must implement appropriate error control methods to minimize or mitigate these errors as effectively as possible. In response to the identified errors, the following two error control methods are proposed.

#### 4.2.1. Improved Installation Accuracy

Prior to installation, employ precise measuring tools (such as measuring tape or laser range finders) to obtain accurate measurements of the installation site. Subsequently, mark the exact locations using marking tools like pencils or chalk. Ensure the use of tools and materials suitable for the working conditions, such as high-quality mounting tools and durable materials, which will improve the accuracy and durability of the installation. Throughout the installation process, conduct periodic checks on the installed components and calibrate them as required. This proactive approach prevents error accumulation and guarantees the continual attention and verification of installation accuracy.

By adhering to these methods, the monitoring system’s accuracy can be maximized to the greatest extent possible.

#### 4.2.2. Error Compensation

To enhance installation accuracy, errors can be compensated for within the host computer program. During the setup phase of the position monitoring system, a wooden board is positioned vertically near the outer wall of the rotor, within the measurement range of the ultrasonic ranging sensor. This setup ensures that the data collected by the sensor represents the horizontal distance from the sensor probe to the rotor’s outer wall. Subsequently, the host computer program subtracts this horizontal distance from the sensor data values. When the ultrasonic sensor data value is zero on the host computer interface, it indicates the horizontal clearance between the rotor and stator during the lifting process. This approach effectively achieves error compensation. The specific compensation formula is outlined as follows.

(33) $$L_{clearances}=L-∆L$$

In the formula: $L_{clearances}$ is the compensated value of the clearance between rotor and stator; L is the actual data output by the sensor; ∆L is the compensation value of the sensor.

By integrating these two methods, we can effectively minimize or even eliminate the impact of all error types within the position monitoring system. This combined approach will significantly enhance accuracy, facilitating a precise fit between the rotor and stator.

## 5. Conclusions

Large components play an increasingly pivotal role in modern industry, owing to their diverse assembly characteristics, often necessitating distinct assembly methods to achieve precise alignment. This paper addresses the challenge of gap monitoring in large components, particularly focusing on the assembly of rotors and stators in hydroelectric generators. A spatial relative position monitoring system is designed for this purpose, emphasizing the inability to construct an external monitoring system.

The position monitoring system is centered around the assembly of the rotor and stator, employing ultrasonic and laser ranging sensors to obtain precise spatial relative position data. A dedicated host computer program resolves these positions, ensuring the accurate alignment of the rotor.

The paper undertakes an extensive error analysis of the system, identifying 24 distinct error sources and employing mathematical modeling to quantify their impact. To mitigate these errors, two primary error control strategies are proposed: enhancing installation accuracy and implementing error compensation techniques. This combined approach effectively minimizes or eliminates various error types within the monitoring system, thereby guaranteeing assembly precision and stability.

In conclusion, the spatial relative position monitoring system and error modeling methodologies proposed in this study provide crucial technical support and assurance for gap monitoring in large component assembly, particularly in the context of assembling large hydro generator rotors. By applying these methods, the traditional difficulty of constructing a monitoring system for seam monitoring in large components has been solved, and the accuracy, reliability, efficiency, and safety of the assembly process have been significantly improved.

## Figures and Tables

**Table 1:** 24 Errors in the position monitoring system.

Types of Errors	East	South	West	North
Positioning	$\delta_{L_{21}},\delta_{L_{31}}$	$\delta_{L_{22}},\delta_{L_{32}}$	$\delta_{L_{23}},\delta_{L_{33}}$	$\delta_{L_{24}},\delta_{L_{34}}$
Measurement	$\delta_{h_{1}}$	$\delta_{h_{2}}$	$\delta_{h_{3}}$	$\delta_{h_{4}}$
Angular	$\varepsilon_{1}$	$\varepsilon_{2}$	$\varepsilon_{3}$	$\varepsilon_{4}$
Perpendicularity	$\theta_{1}$	$\theta_{2}$	$\theta_{3}$	$\theta_{4}$
Calibration	$Z_{1}$	$Z_{2}$	$Z_{3}$	$Z_{4}$

## Data Availability

Data is contained within the article.
